# Supplementary material for: Scale free topology as an effective feedback system
Source: PLoS Comput Biol. 2020 May 11;16(5):e1007825. doi: 10.1371/journal.pcbi.1007825 (PMC7241857; doi:10.1371/journal.pcbi.1007825)
Supplement: Attachment — Submitted filename: Replies_Jan 23_v2.pdf [file pcbi.1007825.s001.pdf]

<u>Reviewer #1:</u> Rivkind et al. submitted an interesting numerical study of the scale-free networks of interacting binary units (Boolean). The authors first show the convergence probability (over finite realizations) to a fixed point is much lower for Scale-Free-Out (SFO) compared with Scale-Free-In (SFI) degree networks. The second result of their paper is the illustration of "lumped hub approximation" for the SFO network. Remarkably, lumped hub approximation captures the distribution of frozen states and convergence to probability to a fixed point. After that, the authors analyze a Mean-Field Theory (MFT) for the lumped hub approximation to give more insight for the effect of hubs on the recurrent dynamics. Here, the authors study two closed and open-loop interactions between the hub and bulk part of the approximate network. Furthermore, they investigate the applicability of the MFT for the open/closed loop approximation.

I believe this is undoubtedly nice work, and it is relevant for the various subfields of computational biology and theoretical neuroscience. Therefore, I support its publication in PLoS CB.

However, in my opinion, the authors have to address three major points in the current manuscript to secure the desired impact:

First, it is unclear to me whether SFO networks converge to point-wise deterministic dynamics. The variance of the column sum in these networks is diverging, and as a result, it breaks the conditions for the existence of a deterministic MFT, as described in Farkhooi and Stannat (PRL, 2017). This has to be discussed in the revision and the notion of a fixed point in this context has to be appropriately defined.

We thank the reviewer for the positive assessment of our work.

Thanks also for referring to Farkhooi and Stannat (PRL, 2017). Their conditions for mean field convergence fail in the scale free setting, highlighting the need for an alternative approximation scheme. This is now added to the introduction. Indeed, these conditions are fulfilled in the auxiliary 'open loop' setting after lumping (eq.2). Our approach may prove useful also for other cases not complying with the conditions, as now stated in the Discussion.

Second, the comparison between the SFO and lumped hub approximation can be improved. I'd suggest two different comparisons in temporal statistics of the networks, and time-averaged properties should be given. This comparison can be used to motivate further analysis in the paper.

Following the reviewer's suggestion, we added two comparisons involving temporal statistics of the networks. First, we computed the participation ratio as a measure of the dimensionality of dynamics in the non-converging case. The correspondence between SFO and lumped-hub approximation is seen in the Figure below, with two stages of coarse graining shown. These data are now added to the revised paper in a new Appendix (Figure S2).

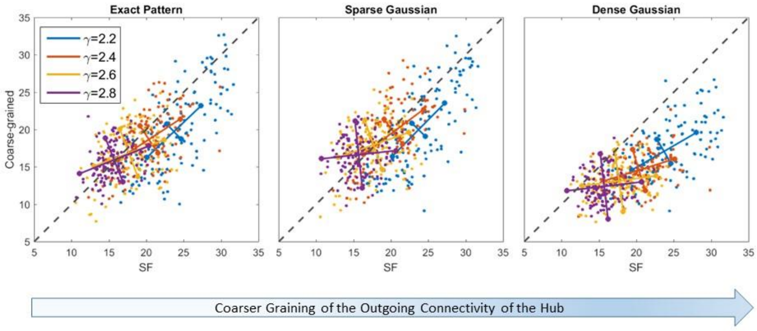

Figure A: Participation ratio computed from dynamic trajectories, for different levels of coarse-graining int the lumped hub approximation.

(Fig. S2 in revised version).

Second, we computed the autocorrelation functions of dynamic trajectories. Like other statistics, they greatly vary from one realization to the next in Scale-Free ensembles. Nevertheless, average correlations functions – also, like other statistics – show a favorable comparison to the corresponding lumped-hub ensemble. This is quantified by the exponential fit to the autocorrelation function. These data are now added to the revised paper in a new Appendix (Figure S3).

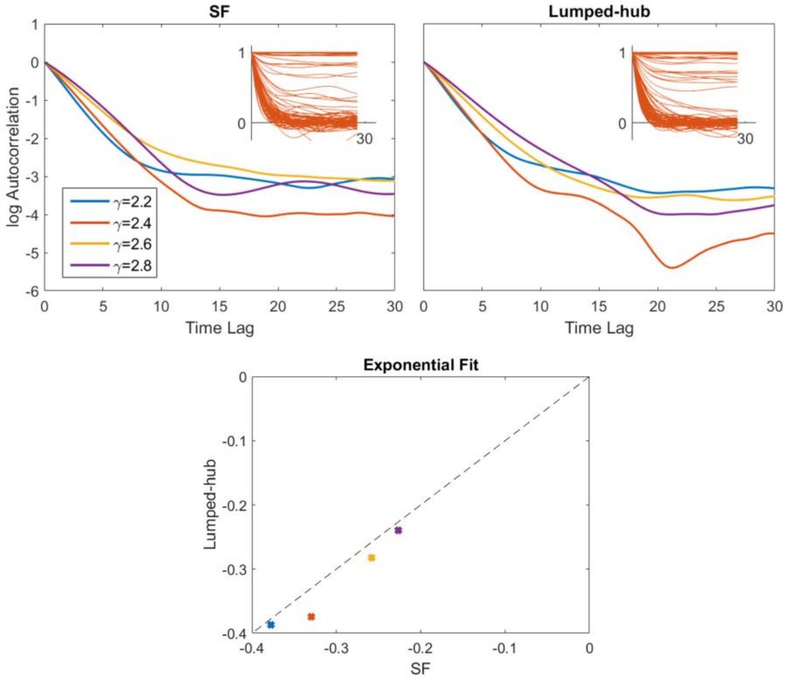

Figure B: Autocorrelation functions of dynamic trajectories in SF and corresponding lumped-hub ensembles. Inset: individual realization in one of the ensembles. Bottom: Exponential fits to the first 5 time units of the autocorrelation functions, providing a quantitative comparison between SF ensembles and their lumped hub approximations.

(Fig. S3 in revised version).

Third, the open/closed-loop analysis using MFT can be improved. It is not so clear for me (in the way that it is written in the main text) if suppression of chaos and the phase transition depends on the approximation itself. I wonder if authors could provide auto-correlations of the system before phase transition for both the approximation and full SFO networks.

We thank the reviewer for highlighting this important point, which perhaps was not formulated clearly enough.

The original SF ensemble does not seem to have a sharp phase transition when varying any of its generating parameters. In contrast, the approximated lumped-hub ensemble does have a phase transition as its effective parameters are varied; the relevant control parameter is identified in our analysis as ∇d. This insight enables us to observe the changing behavior also in the original ensemble, when plotted as a function of the corresponding parameter ∇d (See Fig. 6B).

We now emphasized this point (First paragraph of the section "Applicability of feedback analysis to finite SFO networks" and in Discussion).

Reviewer #2: The authors investigate how the topology determines the controllability of a dynamic system, and investigate specifically the impact of the high-degree nodes in a scale-free topology. Scale-free topologies are characterized by many nodes with only one or a few connections, some nodes with intermediate number of connections, and a few nodes with very many connections. They bundle the highly connected nodes into a single one, and replace the remaining part of the scale-free

network by a random network. This simplification facilitates analytical mean-field treatment. They then show under which conditions the network converges – or does not converge to a fixed point.

I am divided with respect to the impact and generality of the results. On the one hand, it is a very elegant and creative framework, and the results are convincing. On the other hand, the approximations for the mean-field are rough, and the dynamics on the network is very simple, including the fixed-point as an absorbing state. Thus, I am not yet convinced that the framework is of general biological relevance.

Specific comments:

Major:
It remains a question whether the "lumped-hub" approximation is a good one. Taking e.g. figure 4A, there is some correlation, but I guess almost any coarse-graining of the topology would result in some correlation here. Can you argue that your choice of coarse-graining is a good one in general? Specifically, why lumping together four, not three or five of the most connected nodes? Why randomizing the other topology, not just keeping it? How much does it impact the results?

This is indeed a major point in our work, and we thank the reviewer for highlighting and allowing us to sharpen it.

First, we note that as pointed by reviewer #1, SFO networks do not admit a valid mean field approximation. Therefore, it is nontrivial and not a-priori expected that any scheme would work. The difficulty in coarse graining these heterogeneous networks was an important motivation for our work.

In general, we tested several coarse graining strategies and found that not all of them work equally well. The figure below (now included in the paper) follows the effect of increasing levels of coarse graining in the approximation: "Lumped hub" substitutes m hubs by a single one, maintaining its exact connectivity; "Sparse Gaussian" defines an ensemble maintaining the hubs' sparseness and variance of connection strengths; and "Full Gaussian" maintains only the variance, with a hub fully connected to the bulk. The approximation we chose is the Sparse Gaussian, arguably the simplest (or coarsest) approximation that still preserves network behavior.

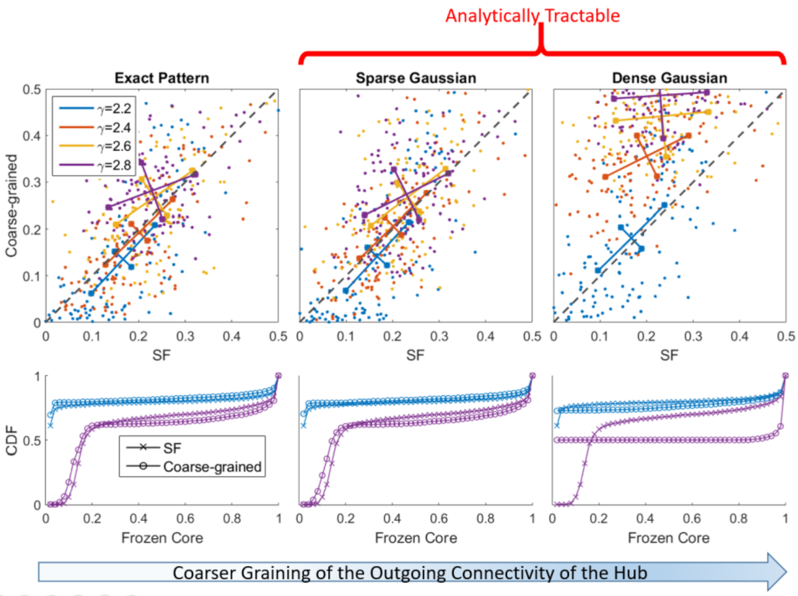

Figure C: Different levels of coarse graining in the lumped-hub approximation. Top: probability of converging to a QFP. Bottom: Cumulative distribution function of frozen corse sizes. Color codes for SF parameters, as indicated in legend.

(Fig. 4 in revised version)

As for the choice of the m=4 largest hubs, this is a compromise between two factors. On one hand, we want to avoid the effect of intra-hub dynamics (which we neglected in the approximation) on the stability analysis. On the other hand a very small value of m, would underestimate the coordinating effect on the bulk network.

Figure 7 and the section on "limits to the lumped hub approximation" explored different choices of m. Figure 7A shows a weak sensitivity to the lumping factor – the change of ~0.1 in ∇d is within the accuracy of our approximation (compare to width of transition region around 0.2 in figure 6B).

Minor:
Abstract: "Based on the observation that in finite networks a small number of hubs have a disproportionate effect on the entire system, we construct an approximation by lumping these nodes into a single effective hub, which acts as a feedback loop with the rest of the nodes."
Only fairly late it becomes clear, why you combine the set of 'most important nodes' to one node, instead of averaging exactly in the opposite manner, namely over all the non-important hubs:
It seems to be much more intuitive, to reduce e.g. all the nodes with degree 1, which is the most frequent degree in a scale-free network, and then potentially also remove the other low-degree nodes, until a treatable topology is found.
It would be good to make the aim more clear at an earlier point.

We thank the reviewer for this viewpoint. Our approximation indeed was aimed at arriving at a treatable topology. This point is now emphasized more clearly in the new Figure 4.

We note that the small-degree nodes do not pose a barrier for a treatable topology. As noted by Revewer #1 above follogin Farkhooi and Stannat (PRL, 2017), it is the large-degree nodes that pose a problem for analysis, and hence were the focus of our approximation.

The terms "source" and "sink" might help the description for the outgoing/ incoming hubs.

Please discuss whether scale-free distributions are really a reasonable description for neural topology: Is it really the case that the most frequent projection pattern of a neuron is to connect to just one other neuron? (p(k=1)>=p(k=N) for all N. – I doubt so.

We agree with this doubt regarding low-k connectivity in neural networks. The scale-free distributions we used has two properties – the power-law tail and the maximum at k=1 mentioned here. Our focus was on lumping the power-law tail; in fact, our approximation does not preserve the monotonic behavior at small k, since we substitute the bulk by a homogeneous distribution. The success of this approximation indicates that the exact nature of the small-k behavior is not crucial for the properties we analyzed.

Equation 1: I'd suggest to write out the sign function.

We have done this in the revised version. Furthermore, this suggestion has highlighted a certain technical point that appears when using a symmetric sign function in sparse networks. We have tested the influence of this point on the results and found it to be negligible. This is now explained in a new Methods section, "Sign function", in the revised version.

Figure 4: I'd suggest to use CDFs instead, and plot both PDFs in a single panel.
Done

Fig. 5: "Mean field theory predicts a phase transition at \sigma_crit".
Could you elaborate about the position of the \sigma_crit? I would have expected it at about \sigma_h=1.2 for the red, and close to zero for the blue condition, i.e. at the point where QFPs start to emerge?
Mean Field predicts a step-function transition between the two phases. For a finite system size we expect this to become a sigmoid with a certain width, with the theoretical prediction roughly in the middle of the smooth gain. Fig. 5B shows that the transition line is slanted in the k-sigma plane, which implies that the width of the transition will be different when traversing k or sigma. We have now added a panel to Fig. 5 showing also the projection of the simulation on the k-axis. It also illustrates the finite-size effect by using an increased system size.

We are aware that approximation worsens as we approach the extremely sparse case ($\sigma_{crit} = 0$ or $\alpha = 0$). The theoretical region of validity is now depicted in the new version of Fig. 5.

An additional change: we realized that figure 6C was not scaled correctly with σ (it was actually plotted against $\sigma\sqrt{\alpha}$). Now fixed.
